# Effect of Different Cooking Methods on Folate Content in Chicken Liver

**DOI:** 10.3390/foods9101431

**Published:** 2020-10-09

**Authors:** Marta Czarnowska-Kujawska, Anna Draszanowska, Elżbieta Gujska

**Affiliations:** 1 Department of Commodity and Food Analysis, The Faculty of Food Sciences, University of Warmia and Mazury in Olsztyn, 10-726 Olsztyn, Poland; elka@uwm.edu.pl; 2 Department of Human Nutrition, The Faculty of Food Sciences, University of Warmia and Mazury in Olsztyn, 10-718 Olsztyn, Poland; anna.draszanowska@uwm.edu.pl

**Keywords:** folic acid, folate, vitamin B, food composition, animal liver, cooking methods, sous-vide, steaming, combi oven, HPLC

## Abstract

Common liver sources in European countries include cow, chicken, duck, lamb and pig. Despite its decreasing popularity, liver is possibly one of the most nutrient-dense foods, being rich in high-quality protein and low in calories. In animals, the liver is the storage organ for folate. In this study, the effect of different cooking methods on folate vitamers content in chicken liver was investigated. Three folate derivatives, 5-CH_3_-H_4_PteGlu, H_4_PteGlu and 5-HCO-H_4_PteGlu, were identified in the analyzed samples using high performance liquid chromatography (HPLC). The folate content in liver after sous-vide (60 °C/75 min) and steaming (100 °C/30 min) did not differ significantly (*p* ≤ 0.05) from raw liver folate content (781 µg/100 g). Even liver cooked in a combi oven or grilled (which resulted in significant folate losses) showed much higher folate content, 455–631 µg/100 g and 612–715 µg/100 g, respectively, than the most folate-abundant plant foods. These findings are important as they demonstrate that processed liver has the potential to improve the supply of folate and meet the recommended daily requirements, particularly when folate deficiency is common worldwide.

## 1. Introduction

B-vitamin folic acid exists naturally in foods in polyglutamyl forms, of which the most common are tetrahydrofolate, 5-methyltetrahydrofolate and 5-formyltetrahydrofolate. Folic acid is the most stable form in terms of chemical degradation and the most bioavailable and therefore is used for both fortification and supplementation purposes [1,2]. Folate vitamers are thought to play a crucial role in the prevention of many diseases, including neural tube defects [3], megaloblastic anemia [4], cardiovascular disease [5] and some types of cancers (colorectal and colon) [6,7]. Other health problems related to common folate deficiency may include neurocognitive decline in the elderly [8]. Due to the increased recognition of the potential protective role of folate in the above-mentioned health problems, the European Food Safety Authority established a Population Reference Intake for folate at the level of 330 µg/day, and of 600 µg/day during pregnancy or lactation (or for women planning pregnancy) [9]. To meet the recommendation, the most folate-deficient groups (pregnant women or the elderly) are advised to folic acid supplementation. Some countries, such as the US and Canada, have introduced mandatory folic acid fortification of cereal-grain products [10]. Since European countries, due to safety concerns regarding excess folic acid intake (for instance, for vitamin B_12_ deficiency in the elderly) [11], are reluctant to introduce obligatory folic acid fortification, alternative approaches to achieve optimal folate status need to be considered [12]. Recent studies promote the consumption of folate-enriched foods like eggs and bread along with the addition of folate-abundant plants [12,13]; moreover, promising but still not popular products, like tropical fruits, have the potential to improve the supply of this critical vitamin [14]. Blancquaert et al. [15] summarized the folate biofortification efforts in crops, lettuce, tomato and potato through metabolic engineering. 

Meanwhile, a rich natural source of folate, but often ignored, is animal liver. The once popular liver has been considered to be unhealthy, high in fat, with carcinogenic potential and it became less common than muscle meats, which now tend to be favored by most consumers. Liver, apart from being cheap and widely available, is also an important source of several micronutrients. An amount of 100 g of cooked beef liver provides more than the RDI (recommended daily intake) for vitamin B_12_, vitamin A, riboflavin and copper. It is also a rich source of choline and iron [16,17,18,19,20]. However, liver is also the storage organ for most folate in the body [21] which results in a large amount of this vitamin in a liver portion compared to most folate-abundant plant origin sources, such as broccoli, spinach or legumes [20,22,23,24,25]. According to Winkles et al. [26], folate derived from liver also has high aggregate bioavailability (approximately 80%), same as that from vegetables and fruit. The previous recommendation to avoid liver consumption has been overturned by recent studies showing bovine liver to be almost free of substances of very high concern such as heavy metals [27,28]. 

The available literature on the folate content in the most common sources of liver (beef, chicken and pork) are scarce and differ widely. For dietary recommendations, it is important to have appropriate information on the availability of nutrients in any food product as well as the effect of different processing methods on their retention. The current study aimed to determine the stability of folate vitamers in chicken liver using validated HPLC methods following preparation with different techniques of culinary treatment such as sous-vide, steaming, combi oven, and grilling.

## 2. Materials and Methods 

### 2.1. Samples

The chicken liver was purchased at the poultry slaughterhouse on the day of slaughter and conducted to different heating treatment as shown in Table 1. For each treatment, 3 portions (3 × 500 g) of fresh rinsed and dried liver were processed. Then samples were blended (Robot Coupe Mini MP 190 v.v.; Montceau-en-Bourgogne, France) and 200 g of sub-sample was taken for the determination of folate content. 

### 2.2. Reagents, Standards and Enzymes

All reagents used were of analytical grade, apart from methanol and acetonitrile which were of HPLC grade. Water was purified using Mili-Q system (Millipore; Vienna, Austria). Folate standards, folic acid (PteGlu), 5-methyltetrahydrofolate (5-CH_3_-H_4_PteGlu), 5-formyltetrahydrofolate (5-HCO-H_4_PteGlu) and tetrahydrofolate (H_4_PteGlu) were obtained from Sigma Aldrich (St. Louis, MO, USA); 10-formylfolic acid (10-HCO-H_4_PteGlu) and 5,10-methenyltetrahydrofolate (5,10-CH^+^-H_4_PteGlu) were obtained from Schircks Laboratories (Jona, Switzerland). Standards were all prepared as described by Konings [29]. 10-formyldihydrofolate (10-HCO-H_2_PteGlu) was obtained from 5,10-methenyltetrahydrofolate according to Pfeiffer et al. [30]. Fresh rat plasma was purchased from Europa Bioproducts Ltd. (Cambridge, Great Britain), α-amylase (E.C.3.2.1.1) and protease (E.C.3.4.24.31) from Sigma Aldrich.

### 2.3. Sample Pretreatment

**Figure 1:**
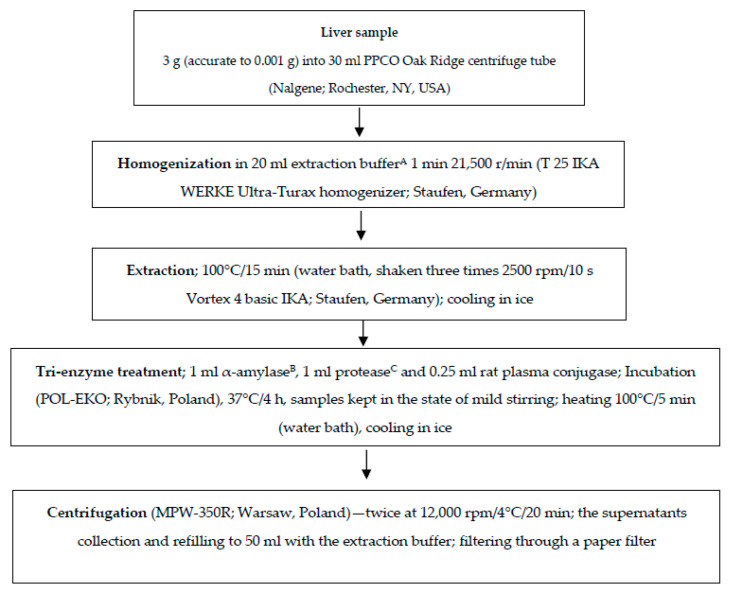
The sample preparation flow chart; ^A^ extraction buffer: 0.1 M phosphate buffer pH 6.1 with 1% (*w*/*v*) sodium ascorbate and 0.1% (*v*/*v*) 2-mercaptoethanol; ^B^ 1 mL α-amylase solution (20 mg/mL); ^C^ 1 mL protease solution (4 mg/mL).

The folate vitamers content was determined with the method described by Czarnowska-Kujawska et al. [25]. Samples were analyzed in triplicate as presented in Figure 1. Folate protection from oxidation during the sample pretreatment was provided by keeping the sample under subdued light, the use of nitrogen, and cooling in ice after heating. Sample extracts purification was carried out before HPLC analysis using Solid Phase Extraction (SPE) on Strong Anion Exchange (SAX) Bakerbond spe. JT cartridges (3 mL × 500 mg Solid Phase Extraction Column, PP (polypropylene), Quaternary Amine (N^+^) Anion Exchange; Philipsburg, USA) as outlined by Jastrebova et al. [31]. Briefly, 4 mL of sample was inserted on preconditioned SAX column and eluted with 4 mL of elution buffer (0.1 M sodium acetate containing 10% (*w*/*v*) sodium chloride and 0.1% (*v*/*v*) 2-mercaptoethanol).

### 2.4. HPLC Analysis

The HPLC separation (Shimadzu Series LC-10A; Shimadzu Co.; Kyoto, Japan) of folate was conducted as described by Czarnowska and Gujska [32]. For separation, a Synergi 4u Hydro-RP 80A (250 × 4.6 mm) 4-µm column was used (Phenomenex; Torrance, CA, USA). The total separation time was 41 min. The gradient elution parameters were as briefly described: injection 50 µL, flow rate: 1 mL/min, column temperature 25 °C, fluorescence detection: 290-nm excitation and 360-nm emission and for 10-HCO-H_4_PteGlu 360-nm excitation and 460-nm emission, UV detection: 290 nm. The mobile phase was 30 mM phosphoric acid buffer, pH 2.3, and acetonitrile. The gradient started with 5% acetonitrile and was maintained as such for the first 8 min until being raised to 17.5% within 17 min. Peaks identification was based on standard retention times. 

Quantification of the identified individual folate vitamers was based on fluorescence detection with the use of external multilevel (*n* = 8) calibration curves, linearity range of 0.3–66.3 ng/mL for 5-CH_3_-H_4_PteGlu (the correlation coefficient > 0.9994), 0.6–55.7 ng/mL for H_4_PteGlu (>0.9996) and 3–150 ng/mL for 5-HCOH_4_PteGlu (>0.9997). The limit of quantification (LOQ) was defined as the lowest analyte concentration yielding a signal-to-noise (S/N) ratio of 10 [31] and were at the level of 0.3, 0.6 and 3.0 ng/mL for 5-CH_3_-H_4_PteGlu, H_4_PteGlu and 5-HCO-H_4_PteGlu, respectively. 

### 2.5. Statistical Analysis

The presented results of folate derivatives content in analyzed samples are all based on the fresh weight (FW) and were demonstrated as means with standard deviations from three repetitions. The given total folate content is presented as the sum of H_4_PteGlu, 5-CH_3_-H_4_PteGlu, and 5-HCO-H_4_PteGlu contents calculated to folic acid with the use of molar absorption coefficient given by Blakely [33]. Differences in the total folate content in raw chicken liver and after different processing methods were compared using Duncan multiple range test, with the significance level at *p* < 0.05. Statistical analysis was carried out using Statistica software version 10.0 (StatSoft; Cracow, Poland).

### 2.6. Method Validation

The results for the validation of the HPLC method for folate determination in liver samples were described in the previous article by Czarnowska-Kujawska et al. [25]. Recovery tests were conducted by analyzing spiked control samples of chicken and pig livers with known amounts of H_4_PteGlu, 5-CH_3_-H_4_PteGlu, and 5-HCO-H_4_PteGlu prior to extraction. Then, the spiked samples were processed through the entire analytical procedure of sample preparation. The recovery (R) was calculated as R = (C_found_ – C_sample_)/C_added_, where C_found_ is concentration in spiked sample, C_sample_ is the concentration in the sample before spiking and C_added_ is the concentration of the added standard. The mean recovery (*n* = 10) was 95% ± 7% for 5-CH_3_-H_4_PteGlu, 91% ± 8% for H_4_PteGlu and 88% ± 9% for 5-HCO-H_4_PteGlu. The repeatability of the analytical treatment was checked with the use of a certified reference material (BCR–487 Pig Liver (vitamins), Joint Research Center; Geel, Belgium) on different extraction days. The obtained total folate value was 12.76 ± 0.61 mg/kg and the mean of 5-CH_3_-H_4_PteGlu was 3.12 ± 0.43 mg/kg. The given results were well in line with the certified amounts of 2.6 mg/kg for 5-CH_3_-H_4_PteGlu and 13.3 ± 1.3 mg/kg for the total folate. 

## 3. Results and Discussion 

The folate content of raw and processed chicken liver obtained with the HPLC method is presented in Table 2. Three folate forms were identified in liver samples: H_4_PteGlu, 5-CH_3_-H_4_PteGlu and 5-HCO-H_4_PteGlu. The methyl form was found to be the dominant folate form in all samples, which is in agreement with previously published data for animal livers [25,34]. The total folate value in raw chicken liver was 781 µg/100 g and was lower than the value previously reported for chicken and turkey raw livers, 1077 and 1078 µg/100 g, respectively, but still higher than for pig and beef raw livers, 554 and 508 µg/100 g, respectively [25]. The obtained result was higher than the total folate content given in the Food Data Central of U.S. Department of Agriculture for raw livers of chicken, 588 µg/100 g, turkey, 677 µg/100 g, and beef, 290 µg/100 g [20].

In the present study, chicken liver samples were prepared using sous-vide cooking, cooking in a combi oven and steaming and grilling with and without an oil addition. Two main mechanisms are described as being involved in folate losses, leaching into the surrounding liquid and oxidation during heat treatment [35]. The effect of different processing methods on folate content using the most common plant origin sources, such as legumes, grain products, green leafy and other vegetables, has been studied in recent years [32,36,37,38,39,40]. Delchier et al. [41] reviewed folate content and stability in fruit and vegetables under most studied processes such as boiling, blanching, steaming, freezing, canning and juicing. High folate losses were observed, inter alia, after canning (from 65% for spinach to 77% for chickpeas), boiling (from 25% for green peas to 70% for chickpeas) and blanching with the highest losses found for spinach (on average from 50% to 95% for hashed spinach). Bureau et al. [39] studied vegetable samples and found no significant folate losses after steaming or microwave heating. None of the thermal treatments applied in the present study caused as drastic folate losses as in the examples of vegetable processing mentioned before and none of the samples had losses exceeding 45%. Moreover, in 5 out of 8 applied treatments, the total folate loss did not exceed 25%. Sous-vide and the combi oven cooking method deserve special attention since there is a lack of data on the effect of these increasingly popular cooking techniques on the folate contents in both plant and animal origin samples. In the sous-vide technique, meat is vacuum-packaged and subjected to heating in a water bath at temperatures lower than in traditional heating treatment. Multi-layer, non-gas-permeable plastic packaging, which is also resistant to high temperature, minimizes the loss of nutrients and water and protects vitamins from degradation during exposure to high temperatures [42]. Moreover, it enables obtaining more juicy, tender and flavorful meat and fish products than high-temperature cooking methods [43,44]. In the current study, sous-vide cooking performed at a lower temperature (60 °C), but for a longer time (75 min), did not cause significant (*p* < 0.05) folate losses compared with raw liver (Table 2). However, raising the temperature to 75 °C and reducing the time of sous-vide cooking to 45 min resulted in a significant (16%) loss of folate. Steaming performed at 100 °C for 30 min (higher than in the sous-vide method) did not cause significant folate reduction. Steaming was conducted in the oven chamber by steam injection. This technique is increasingly being used in meat processing since it reduces the time of thermal treatment and provides juicier meat products by protecting the product from dehydration of the surface compared to heating in dry air [45,46,47]. Peterson [48] studied the influence of sous-vide processing, steaming and boiling on folate retention in broccoli florets and observed higher folate losses (89% retention of folate in sous-vide compared with 59% for steaming and 25% for boiling, all at 100 °C/40 min).

A combination of moist heat, dry heat and dry heat separately were used in other treatments using the combi oven cooking method. This relatively new cooking technique, which uses a single piece of equipment, can replace cooking needs for a steamer, grill and convection oven. Its increasing use in foodservice results from reduced cooking time and cooking loss, yielding a juicier product with good control of relative moisture and cooking climate. It preserves food quality, including appearance, flavor and nutrients [49]. In the current study, all treatment using hot, dry heat caused significant folate losses. The highest folate reduction (more than 40%) was observed when 100% dry air at 180 °C was applied for 30 min. Lowering the temperature to 160 °C and then applying 50% relative humidity for the remaining 30 min of cooking time resulted in a lower loss of folate (30% less). Only 20% reduction was observed when the cooking time was reduced to 20 min and the temperature was increased to 180 °C and relative humidity was at the level of 30%. The obtained results for the combi oven cooking method indicate that the heating time might be a more important factor (in terms of folate in chicken liver) than temperature change (160–180 °C) and different relative humidity proportions applied (0–50%). In grilled chicken liver, a higher grilling temperature (200–220 °C for 4 min) resulted in lower folate losses of only 8% compared with grilling with an oil addition (22%). Oil-free treatment thanks to shorter grilling time, resulted in less leaching of ingredients and less water evaporation. In the second grilling treatment, the addition of oil caused a lower temperature (170–200 °C) and extended the grilling time to 6 min and resulted in higher folate losses (22%). A previous study by Aramouni and Godber [50] on folate stability in beef liver under cooking and frozen storage, showed higher folate losses after grilling and frying (41% and 50%, respectively). 

The results presented in Table 2 were also used to analyze the stability of individual folate vitamers in chicken liver samples due to the applied heating method. The current observations are in agreement with the findings of other authors summarized by Delchier et al. [41], that different folate vitamers have markedly different stabilities, with H_4_PteGlu to be the least stable under different processing conditions. In the current study, the H_4_PteGlu content in raw liver was 243 µg/100 g. The lowest reduction of this folate form (not exceeding 60%) was observed in sous-vide (75 °C/45 min), steaming and combi cooking with hot air (180 °C) and a relative humidity of 30% for 20 min. In other cooking methods, the losses ranged from 80% to 99%. The 5-CH_3_-H_4_PteGlu initial content of 505 µg/100 g in raw liver was only slightly reduced by no more than 10% in sous-vide (75 °C/45 min), combi cooking of 180 °C and relative humidity of 30%/20 min and combi cooking of 160 °C and a relative humidity of 50%/30 min. Combi cooking with 100% hot air of 180 °C for 30 min caused a methyl folate form reduction of nearly 30%. In the liver samples grilled with an oil addition grilled and steamed and cooked with sous-vide (60 °C/75 min), 5-CH_3_-H_4_PteGlu increased from 10%, in grilled with oil, to nearly 35% in the sous-vide method. Vahteristo et al. The authors in [22] found an increase (up to 136%) in the 5-CH_3_-H_4_PteGlu content in rainbow trout sample after heating. This could result from enzymatic interconversions in fresh liver while preparing the sample for analysis or during the methylation reaction while frying [22]. For 5-HCO-H_4_PteGlu, which was the least present form in raw liver (57 µg/100 g), its content reduction was observed only after sous-vide treatment conducted at 60 °C for 75 min. After other processing methods, an unexpected increase of up to 65% in combi oven cooking with hot air of 160 °C and relative humidity of 50% for 30 min was observed. The explanation for this could be the higher stability of 5-HCO-H_4_PteGlu compared with H_4_PteGlu and 5-CH_3_-H_4_PteGlu under different conditions and spontaneous interconversion, either in the food or during analysis [41]. 

In general, both raw and processed chicken livers were characterized by very high folate content. The current results indicate that even liver samples with significant folate losses (as a result of the applied heat treatment) still had much higher folate content compared to commonly consumed natural folate sources, mainly of plant origin. Table 3 presents a comparison of the results of folate content for processed chicken liver with the literature data for examples of various food samples.

## 4. Conclusions

The present study provides a better understanding of the effect of different cooking methods on folate content in animal liver, confirming the different stability levels of various folate vitamers. Both raw and processed chicken liver were shown to be rich natural sources of folate. The current study found that the consumption of the processed liver should be encouraged, especially due to common folate deficiency and controversy surrounding excess synthetic folic acid consumption. Additionally, in terms of food preparation, sous-vide (60 °C/75 min), steaming and grilling can be recommended for liver preparation in households or food services as an ideal way to maintain maximum folate retention. 

The obtained results inspire further research to provide information on the effect of the different cooking methods on other quality parameters of different animal livers such as lipid profile, cholesterol, color, lipid oxidation or another bioactive ingredients content in order to evaluate the overall effect on the product.

## Figures and Tables

**Table 1:** Heat treatment parameters of liver samples.

Heat Treatment	Description	Parameters (Temperature/Time and Humidity)
1. Sous-vide	Portions of liver were packaged individually in plastic bags with the use of the Busch Edesa vacuum sealer (Montcada, Spain). The bags were made of polyamide/polyethylene (PA/PE) with a thickness of 52 µm, and they were designed specifically for sous vide cooking (Hendi, Austria). The samples were cooked in fusionchef Diamond Z immersion circulator sous-vide cooker by Julabo (Seelbach, Germany).	60 °C/75 min
2. Sous-vide		75 °C/45 min
3. Oven, hot air and steam	Cooked in combi oven (Retional SCC WE 101; Landsberg, Germany).	hot air of 180 °C and relative humidity of 30%/20 min
4. Oven, hot air and steam		hot air of 160 °C and relative humidity of 50%/30 min
5. Oven, hot air		100% hot air of 180 °C/30 min
6. Oven, overheated steam	Steamed with overheated steam in combi oven (Retional SCC WE 101; Landsberg, Germany).	100 °C/30 min
7. Grill	Grilled without oil addition using Roch Guss (Italy) grill pan	200–220 °C/4 min
8. Grill, oil addition	Grilled with rapeseed oil addition using Roch Guss (Italy) grill pan	170–200 °C/6 min

**Table 2:** Folate content in raw and processed chicken liver (µg/100 g fresh weight (FW)).

Method of Heat Treatment	H_4_PteGlu	5-CH_3_-H_4_PteGlu	5-HCO-H_4_PteGlu	Total Folate (as Folic Acid)
Raw sample	243 ^1^ ± 23	505 ± 41	57 ± 4	781 ± 64 ^a 2^
Sous-vide 60 °C/75 min	48 ± 2	677 ± 13	51 ± 1	749 ± 14 ^ab^
Sous-vide 75 °C/45 min	126 ± 10	490 ± 13	60 ± 1	654 ± 18 ^c^
Oven, 180 °C/20 min, 30% humid.	98 ± 6	482 ± 18	73 ±3	631 ± 24 ^c^
Oven, 160 °C/30 min, 50% humid.	4 ± 1	457 ± 37	94 ± 7	534 ± 41 ^d^
Oven, 100% hot air, 180 °C/30 min	11 ± 1	371 ± 27	92 ± 10	455 ± 26 ^e^
Oven, steam 100 °C/30 min	100 ± 5	627 ± 10	82 ± 5	780 ± 17 ^a^
Grill 200–220 °C/4 min	3 ± 1	657 ± 9	83 ± 6	715 ± 12 ^b^
Grill, oil addition 170–200 °C/6 min	11± 1	556 ± 37	69 ± 5	612 ± 32 ^c^

^1^ The results are presented as the mean of three replicates ± standard deviation. ^2^ Means with the same letter are not significantly different at *p* < 0.05.

**Table 3:** Folate content in raw and processed food samples (µg/100 g FW).

Food Sample	Folate Content	Reference
Processed chicken liver	455–780	Own study
Butterhead lettuce	71	[51]
Rocket in protective atmosphere, rinsed	198	
Fresh broccoli	159	[32]
Frozen broccoli	143	
Fresh cauliflower	89	
Frozen cauliflower	82	
Raw French bean	132	[52]
Boiled French bean	48	
Faba beans dried	96	[53]
Faba beans canned	18	
Raw chickpeas	226	[38]
Soaked chickpeas	343	
Blanched chickpeas	271	
Baker’s yeast	874	[13]
Wheat flour	48	
Fresh spinach	238	
40 g/100 g spinach fortified whole-grain bread	117	
Fresh rye bread	83	[36]
Raw whole fruit guavas (tropical fruit)	91	[14]
Jack fruit chips (tropical fruit)	192	
Banana	13	[54]
Strawberry	80	[53]
Sweet potato	22	
Raw folate-enriched eggs	135	[12]
Folate-enriched eggs after 3 min boiling	125	
